# The Association between Grand Multiparity and Adverse Neonatal Outcomes: A Retrospective Cohort Study from Ha’il, Saudi Arabia

**DOI:** 10.3390/children10091541

**Published:** 2023-09-12

**Authors:** Hend Alkwai, Farida Khan, Reem Alshammari, Asma Batool, Ehab Sogeir, Fahaad Alenazi, Khalid Alshammari, Ayesha Khalid

**Affiliations:** 1 Department of Pediatrics, College of Medicine, University of Ha’il, Ha’il 55473, Saudi Arabia; 2 Department of Family and Community Medicine, College of Medicine, University of Ha’il, Ha’il 55473, Saudi Arabia; fa.khan@uoh.edu.sa (F.K.); refalshammari@uoh.edu.sa (R.A.); e.sogeir@uoh.edu.sa (E.S.); 3 Department of Obstetrics and Gynecology, Maternity and Children Hospital, Ha’il 55471, Saudi Arabia; drasmafaisal@yahoo.com; 4 Department of Pharmacology, College of Medicine, University of Ha’il, Ha’il 55473, Saudi Arabia; fs.alenazi@uoh.edu.sa; 5 Department of Medicine, College of Medicine, University of Ha’il, Ha’il 55473, Saudi Arabia; kf.alshammari@liveuohedu.onmicrosoft.com; 6 William Harvey Hospital, Ashford TN24 0LZ, UK; ayesha-a.khalid@nhs.net

**Keywords:** parity, grand multiparity, neonatal outcomes, Ha’il, Saudi Arabia

## Abstract

Inconsistent evidence exists regarding the association of grand multiparity with adverse neonatal outcomes. This study aims to compare specific adverse outcomes in grand multiparas (those with five or more births at twenty or more weeks of gestation, regardless of fetal outcome) compared to those with lower parity (those with less than five births at twenty or more weeks of gestation, regardless of fetal outcome). A retrospective cohort study was undertaken at the Maternity and Children Hospital in Ha’il region, Saudi Arabia. After calculating the required sample size, data were collected from consenting participants with a viable singleton delivery. Socio-demographic variables, select maternal characteristics, and adverse neonatal outcomes (admission to the neonatal intensive care unit, low birth weight, prematurity, and APGAR score less than 7 in the first 5 min) were compared between grand multiparas and women with lower parity. Two hundred ninety-four participants were recruited (ninety-eight grand multiparas and one hundred ninety-six of lower parity). There was a statistically significant difference between the two groups in relation to age, level of education, body mass index, and the occurrence of gestational diabetes. Out of the studied adverse neonatal outcomes after the adjustment for maternal age between the two groups, no statistically significant difference in the adverse neonatal outcomes was found between the two groups. Grand multiparity does not incur an additional risk of adverse neonatal outcomes compared to women of lower parity. Furthermore, increasing maternal age and comorbid conditions might have a more detrimental effect on neonatal outcomes than grand multiparity per se.

## 1. Introduction

The terminology used to define a woman’s parity has been a topic of debate within the clinical community [1,2]. While a consensus has yet to be reached, there is a generally accepted definition of parity that considers the number of pregnancies in which a woman gives birth at twenty or more weeks of gestation, regardless of the outcome (live or stillborn) or the number of fetuses (counting twins as a single event) [3,4]. Similarly, the definition of grand multiparity has been a subject of disagreement, as it has evolved over time to reflect changes in fertility rates. In recent decades, grand multiparity has generally been defined as a woman having five or more births at twenty or more weeks of gestation, irrespective of the outcome of each pregnancy. On the other hand, the term “great grand multipara” is used to describe women who have had ten or more births (live or stillborn) at twenty or more weeks of gestation. This adjustment in the definition acknowledges the shifting reproductive patterns and ensures that the term grand multiparity remains relevant and consistent with the current demographic landscape. The reported prevalence of grand multiparity varies across countries, reflecting differences in population demographics. Additionally, variations in the definition of grand multiparity, as well as differences in study settings and designs, contribute to the discrepancies in reported prevalence rates.

Over the past five decades, there has been a consistent global decline in the total fertility rate (TFR). The TFR represents the average number of children that a woman would have in her lifetime if the observed fertility rates at each age remained constant. This decline in TFR reflects a broader global trend of decreasing fertility rates [5]. It is important to note that there is also a notable disparity in TFR between high-income countries and low- and middle-income countries. Generally, high-income countries tend to have lower TFRs compared to their counterparts in low- and middle-income countries. According to data from The World Bank for Saudi Arabia in 2021, the reported TFR is 2.4. Indeed, it is essential to highlight the wide range of TFR observed across participating countries, which exemplifies the diverse global landscape of fertility rates. The TFR range spans from as low as 0.8 up to 6.8 in the report from 2021, highlighting the significant variations in reproductive patterns and preferences worldwide. This diversity underscores the complex interplay of social, cultural, economic, and demographic factors that shape fertility rates and contribute to the unique fertility profiles observed in different countries.

The exploration of potential complications faced by multiparous women has been a subject of inquiry dating back to as early as 1934 [6]. In the influential work by Bethel Solomons, an extensive exploration of potential obstetrical complications faced by grand multiparas is presented. These complications encompass various aspects, including a pendulous abdomen, eclampsia, hemorrhage, malpresentation, placenta previa, adherent placenta, and even the rare occurrence of a ruptured uterus. Solomons sheds light on these challenges, emphasizing the importance of understanding and addressing them to ensure optimal maternal health and well-being. Neonatal complications associated with grand multiparity have also been extensively described, notably including prematurity and low birth weight [7,8,9,10,11], neonatal intensive care unit admission [9,12,13], and perinatal death [11,13,14].

As the literature on grand multiparity expanded, conflicting conclusions emerged regarding the obstetric and neonatal complications associated with this condition. These discrepancies can be attributed to several factors that warrant consideration. Firstly, variations in the definitions of cases and control groups employed across studies may contribute to inconsistent findings. The lack of standardized criteria for classifying grand multiparity and the inclusion of different comparison groups can introduce variability in the reported outcomes. Furthermore, inadequate adjustment for confounding variables, such as maternal age, can also impact the interpretation of study results. Maternal age is known to be associated with both grand multiparity and adverse pregnancy outcomes, and failing to account for this factor adequately may lead to confounding effects and misleading conclusions. Additionally, disparities in healthcare access, provision, and quality can influence the observed outcomes among grand multiparous women. Variations in healthcare systems, availability of prenatal care services, and socio-economic factors may contribute to differences in the management and outcomes of pregnancies.

Given these complexities, it is imperative to cautiously approach the literature on obstetric and neonatal complications associated with grand multiparity. Further research that addresses these limitations and employs robust methodologies is needed to gain a more comprehensive understanding of this topic and guide clinical practice effectively. There is a paucity of literature concerning the neonatal outcomes of grand multiparous women in Saudi Arabia, particularly within the specific context of Ha’il region. Recognizing this research gap, the primary objective of this study is to shed light on this topic by conducting a comprehensive examination of individual adverse neonatal outcomes. Specifically, we will investigate the rates of admission to the neonatal intensive care unit, cases of low birth weight, instances of prematurity, and infants with an APGAR score below 7 at 5 min. By comparing these outcomes between grand multiparous women and those with lower parity (ranging from one to four births), we aim to contribute valuable insights to the existing knowledge in this field.

## 2. Materials and Methods

### 2.1. Study Region and Period

Ha’il is one of the thirteen administrative regions (also called provinces or emirates) in Saudi Arabia (S.A.). It is located in the northern part of S.A., around 645 km from the capital city of Riyadh. Ha’il is the eighth-largest region by area. The estimated population is 731,147, making it the ninth most populous region. Furthermore, Ha’il is subdivided into nine governorates, with more than two-thirds of the population living in the main Ha’il governorate. This study was conducted at the Maternity and Children Hospital, the only Ministry of Health (MOH) public hospital providing perinatal services in the main Ha’il governorate. It has both an obstetric and neonatal intensive care unit. Data collection commenced in October 2021 and continued until the calculated sample size was reached.

### 2.2. Study Design and Recruitment

This was a retrospective cohort study. The population of interest was grand multiparas who had given birth at the hospital. Grand multiparas (patients with five or more births at twenty or more weeks of gestation, regardless of fetal outcome) were the exposure group. On the other hand, women with lower parity (patients with at least one but less than five births at twenty or more weeks of gestation, regardless of fetal outcome) were enrolled in this study as the nonexposed group. Due to difficulty documenting perinatal outcomes, women who delivered at home before admission and those transferred from other hospitals after delivery were excluded. Excluded were women with conditions associated with a high-risk pregnancy [15], including those with pre-existing chronic medical conditions, including chronic infections, women with certain obstetrical conditions such as multiple pregnancies (pregnancies with more than one fetus) in the current pregnancy, and those with a history of substance abuse. Chronic medical conditions include hypertension, diabetes mellitus, and chronic thyroid, cardiovascular, or renal disease. These chronic diseases were found to increase maternal morbidity and mortality in different studies [16,17]. To ensure a homogeneous study population in terms of obstetric history and to eliminate potential confounding factors related to their unique characteristics and experiences as first-time mothers, primiparas (patients who have not delivered previously) were excluded. Finally, women who were critically ill, unable to communicate, or unwilling to provide their consent were excluded.

### 2.3. Sample Size Calculation and Sampling Technique

Epi Info™ 7.2 (CDC, Atlanta, GA, USA) was used to calculate the sample size. To estimate the sample size, the following parameters were used: a confidence interval of 95%, study power (1-β) of 80%, and a 2:1 ratio for the unexposed-to-exposed group. In addition, the frequency of 8.5% for neonatal low birth weight in the nonexposed group was used from a previous study [7]. The Fleiss formula with Continuity Correction was used for the calculation. After adding 10% for possible nonresponse, the final sample size was 294 (98 for grand multiparas and 196 for women with lower parity). The recruitment of participants was through convenience sampling.

### 2.4. Data Collection and Study Variables

The data collection tool was prepared after the review of similar published studies [7,18,19,20]. Data collectors were trained prior to the actual data collection, and the tool was piloted on 15 participants. Adverse neonatal outcomes of interest included the presence of one or more of the following: neonatal intensive care unit (NICU) admission regardless of cause or duration, prematurity defined as birth prior to 37 weeks of pregnancy, low birth weight defined as birthweight less than 2500 g regardless of gestational age, and an APGAR score less than seven at five minutes after birth. This APGAR cutoff score was used in similar studies as a marker of adverse neonatal outcomes [7,20,21]. Calculation based on the last menstrual period (LMP) was used to estimate the gestational age (G.A.). Ultrasound measurement was used for G.A. estimation when the LMP was not known. In addition, patients’ socio-demographic factors, body mass index (BMI), perinatal health services utilization, certain pregnancy-related health conditions, as well as the mode of delivery were collected as potential confounding variables. The educational level was categorized as no formal education, primary (including elementary and middle school), secondary (including high school), and tertiary (including all post-secondary schooling). Employment status was described as employed (with a regular source of income, including self-employment) and nonemployed.

### 2.5. Data Analysis

The data were analyzed using IBM SPSS Statistics Software, Version 29. Data were cleaned for missing values and inconsistencies. Descriptive statistics such as frequency tables were used to summarize the study variables. An independent-sample t-test was run to compare the means of continuous variables (age and BMI). For these variables, boxplots were inspected for outliers, Shapiro–Wilk test was used to assess the normality of distribution, and Levene’s test was used to assess the equality of variances. To determine if differences existed between the two proportions (grand multiparas and women of lower parity), the test of two proportions was used for dichotomous variables, the nonparametric Pearson chi-square (chi-square test of homogeneity) for categorical variables and the Mann–Whitney U test for ordinal variables. The multivariable logistic regression model was used to calculate the odds ratio (OR) and the 95% confidence interval (CI) to assess adverse neonatal outcomes associated with grand multiparity, namely admission to the neonatal intensive care unit, cases of low birth weight, instances of prematurity, and infants with an APGAR score below 7 at 5 min. Statistical significance was asserted when the *p*-value was <0.05.

### 2.6. Ethical Considerations

The Research Ethics Standing Committee at the University of Ha’il approved this study (approval code H-2022-010). Per the Declaration of Helsinki and after describing this study’s objectives, benefits and risks of participation, and the data collection procedure, written informed consent was obtained from all consenting participants. Participation in this study was voluntary, and no incentives were provided.

## 3. Results

A total of 294 participants were recruited, with 98 grand multiparas and 196 women of lower parity. The age range of participants was 19 to 48 years. Table 1 shows the socio-demographic characteristics of the participants. Grand multiparas’ mean age was six years higher, 95% CI [4.9 to 7.1] compared to those with lower parity. There was a statistically significant difference in the mean age between the two groups, t (241.3) = 10.8, *p* < 0.001. There was also a statistically significant difference in the educational level (*p* = 0.028).

In Table 2, select clinical characteristics of the study population are shown. Grand multiparas’ BMI was greater by 4 kg/m^2^, 95% CI [2.5 to 5.5] compared to those with lower parity. There was a statistically significant difference in the mean BMI between the two groups, t (285) = 5.2, *p* < 0.001. Gestational diabetes was more prevalent among grand multiparas (*p*-value < 0.05).

**Figure 1:**
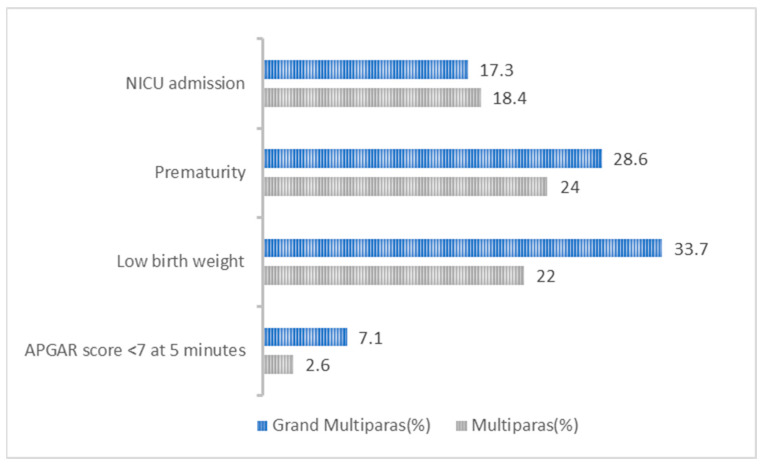
Clustered bar chart for the comparison of neonatal outcomes between the deliveries of grand multiparas and those of lesser parity, Ha’il, SA.

Figure 1 is a clustered bar chart comparing adverse neonatal outcomes between the two groups. To ascertain the effects grand multiparity has on the selected neonatal outcomes, a logistic regression test was performed to calculate the crude odds ratio (COR) and as maternal age is an important confounding factor, we calculated the adjusted odds ratio (AOR) adjusting for maternal age (see Table 3). In that model, grand multiparity was significantly associated with low birth weight (LBW) (OR = 1.806, 95%CI [1.054, 3.095]) (*p* = 0.031) However, after adjustment for maternal age, no statistically significant difference in neonatal outcomes exists between grand multiparas and women of lower parity.

## 4. Discussion

Throughout the years, the definition of grand multiparity has undergone adjustments in response to the declining total fertility rates. In the 1960s, the term primarily encompassed women who had experienced eight or more viable pregnancies [22]. However, as societal norms and demographic patterns shifted, the definition evolved. By the 1990s, grand multiparity came to include those who had undergone six or more viable pregnancies [23]. In more recent times, the definition has been further refined to encompass women who have had five or more births at twenty or more weeks of gestation, irrespective of fetal outcome. This changing definition reflects the dynamic nature of demographic patterns and societal perspectives on fertility and childbirth.

The phenomenon of grand multiparity exhibits considerable variation in reported prevalence worldwide, reflecting the diverse contexts in which it occurs as well as the varying trends in the total fertility rates (TFR). The TFR trends mirror variations in socio-economic factors, cultural norms, access to healthcare, and family planning practices across different regions and income levels. Cultural norms encompass societal beliefs, values, and practices related to reproduction and family planning. These norms can vary across cultures and regions, shaping individuals’ attitudes and behaviors toward childbearing. Access to healthcare also plays a crucial role in shaping fertility rates. The availability and affordability of contraceptive methods, family planning services, and reproductive healthcare can empower individuals to make informed decisions about their reproductive choices. Adequate access to healthcare can enable individuals to plan pregnancies, space births, and limit family size according to their preferences and circumstances. Furthermore, healthcare services that address maternal and child health, provide prenatal and postnatal care, and support infertility treatments can influence fertility rates. Improved access to healthcare, particularly in low- and middle-income countries, can contribute to declining fertility rates by reducing infant and maternal mortality, promoting women’s health and education, and increasing the availability of family planning services.

The prevalence of grand multiparity rates exhibits significant variability across different regions and countries. For instance, in the United States, the prevalence is reported to be as low as 2.97% [23], while certain regions in Africa have reported rates as high as 27%. Within the specific context of Saudi Arabia, the reported prevalence of grand multiparity falls within a range of 5.3% [8] to 10.2%, underscoring the variability even within a single country. Neighboring countries with similar socio-economic characteristics, such as the United Arab Emirates, have also reported comparable prevalence rates, with a recorded prevalence of 7.4% [24]. These figures highlight the influence of a multitude of factors, including social, economic, religious, and cultural dynamics, on the prevalence of grand multiparity. These factors shape the reproductive choices and patterns of individuals within specific populations, resulting in the observed variations in prevalence rates. Recognizing and understanding these variations is crucial for the development of targeted interventions and healthcare strategies that effectively address the unique needs and challenges faced by grand multiparous women in different regions.

In the early 1930s, significant attention was drawn to the potential risks associated with multiparity, particularly in comparison to primiparity, by Bethel Solomons, who published a pioneering paper on the subject [6]. Solomons observed adverse obstetrical risks faced by multiparous women, noting a gradual increase in maternal mortality rates after the fifth child. However, when it comes to investigating the effects of grand multiparity on obstetric and neonatal adverse events, the research community has yet to reach a consensus. The lack of consensus in this area can be attributed to various factors. Inconsistencies in the operational definitions of study variables, variations in study designs and statistical approaches, as well as disparities in healthcare access and availability, contribute to the divergence of findings among studies. Additionally, the confounding factor of older age among grand multiparous women plays a significant role in the observed outcomes. However, when adjustments are made for age, along with its associated comorbidities, studies often report similar outcomes between grand multiparas and women of lower parity [9].

In this study, our primary aim was to investigate the potential association between grand multiparity and adverse neonatal outcomes within our specific study population. To achieve this, we conducted a comprehensive comparison of select neonatal outcomes between grand multiparas and women of lower parity. It is important to note that the two groups exhibited similar socio-demographic characteristics for the most part. However, it is worth highlighting that grand multiparas had a statistically significant higher mean age (*p* < 0.001), which is consistent with findings from other cohort studies conducted in Saudi Arabia [7,8,25]. Furthermore, we observed a difference in educational attainment between the two groups (*p* = 0.028), with younger women of lower parity achieving higher levels of education. This difference in educational attainment has also been reported in a previous study conducted in Saudi Arabia [7]. However, it is intriguing to note that a study comparing multiparas to grand multiparas, stratified by age, found no significant difference in educational level when analyzing the subgroup of young grand multiparas (below 35 years of age) [9]. These findings provide valuable insights into the complex interplay between educational attainment, age, and grand multiparity within the specific context of our study population [9].

Clinical characteristics were carefully examined and compared between the two groups under investigation. It was observed that grand multiparas had a higher mean body mass index (BMI) compared to women of lower parity, with this difference being statistically significant (*p* < 0.001). This finding aligns with a previous study conducted by Al-Shaikh et al., which also reported a higher BMI among grand multiparas [7]. Furthermore, the rate of gestational diabetes mellitus (GDM) was found to be higher among grand multiparas in our study. This finding is consistent with the results reported by Alhainiah et al., who similarly described a higher rate of GDM among grand multiparas [25]. These findings collectively suggest a potential association between grand multiparity, higher BMI, and an increased risk of developing GDM.

Four specific adverse neonatal outcomes were chosen for comparison between the two groups: admission to the neonatal intensive care unit (NICU), prematurity, low birth weight, and an APGAR score below 7 at 5 min. Logistic regression was employed to analyze the data and calculate the odds ratio. In this model, grand multiparity was found to be associated with a decreased likelihood of delivering a low-birth-weight infant, with an odds ratio of 0.554 and a 95% confidence interval of [0.323–0.948] (*p* = 0.031). However, when adjusting for maternal age, the statistical significance of the association between grand multiparity and low birth weight became insignificant. This adjustment was implemented due to the significant difference observed in the mean age of the two groups, suggesting the presence of a potentially influential confounding factor. Notably, other studies have reported similar findings, indicating no increase in adverse neonatal outcomes when grand multiparas were stratified into different age groups [9].

## 5. Conclusions

The findings of our study provide valuable support for the notion that grand multiparity is unlikely to result in adverse neonatal risks. Instead, it suggests that factors such as advancing maternal age and the presence of comorbid conditions may have a more substantial impact on neonatal outcomes. These findings have important implications for the care and management of grand multiparous women, who have often been the subject of controversy. Based on our findings, we strongly recommend the implementation of a large-scale prospective study to thoroughly explore the prevalence of grand multiparity in Saudi Arabia. Such a study should also investigate the contributing factors and assess the associated adverse maternal and neonatal outcomes. Ideally, this research endeavor should encompass a national scope to provide a comprehensive understanding of the situation. The insights gained from these research efforts will be instrumental in guiding the development of effective care strategies, and management approaches for this specific population. By shedding light on the prevalence and associated factors of grand multiparity, we can address the controversies surrounding this topic and ensure the provision of optimal healthcare for these women and their newborns.

## Figures and Tables

**Table 1:** Socio-demographic characteristics of the study population.

Variable	Grand Parity	Low Parity	*p*-Value ^§^
	*n* (%) = 98 (33.3)	*n* (%) = 196 (66.6)	
Maternal age (years)			
Mean (S.D.)	39.5 (±4.0)	32.5 (±5.2)	<0.001
Age range (years)			<0.001
≤20	0 (0)	2 (1.0)	
21–25	0 (0)	15 (7.7)	
26–30	3 (3.1)	55 (28.1)	
31–35	18 (18.4)	69 (35.2)	
36–40	49 (50.0)	42 (21.4)	
≥40	28 (28.6)	13 (6.6)	
Educational level			0.028
No formal education	16 (16.3)	15 (7.7)	
Primary	13 (13.3)	14 (7.1)	
Secondary	64 (65.3)	155 (79.1)	
Tertiary	5 (5.1)	12 (6.1)	
Employment status			1.000
Employed	13 (13.3)	25 (12.8)	
Nonemployed	85 (86.7)	171 (87.2)	
Income level (SAR)			0.130
≤4000	4 (4.1)	16 (8.2)	
>4000–8000	61 (62.2)	97 (49.5)	
>8000–12,000	27 (27.6)	61 (31.1)	
≥12,000	6 (6.1)	22 (11.2)	

SD: standard deviation; SAR: Saudi Arabian Riyal. ^§^ Statistical significance was set at a *p*-value < 0.05.

**Table 2:** Select clinical characteristics of the study population.

Variable	Grand Parity	Low Parity	*p*-Value
	*n* (%) = 98 (33.3)	*n* (%) = 196 (66.6)	
BMI at delivery (kg/m^2^)			
Mean (S.D.)	37.3 (±6.1)	33.3 (±6.1)	<0.001
Booking status			
Booked	67 (35.3)	123 (64.7)	0.343
Unbooked	31 (29.8)	73 (70.2)	
Comorbidities			
PIH	8 (38.1)	13 (61.9)	0.631
GDM	26 (53.1)	23 (46.9)	0.001
Anemia	27 (33.3)	54 (66.7)	1.000
Mode of delivery			
Vaginal delivery	48 (34.3)	92 (65.7)	0.530
Assisted vaginal delivery	3 (20.0)	12 (80.0)	
Caesarean section	47 (33.8)	92 (66.2)	

BMI: body mass index; PIH: pregnancy-induced hypertension; GDM: gestational diabetes mellitus.

**Table 3:** Comparison of neonatal outcomes between the deliveries of grand multiparas and those of lesser parity, Ha’il, SA.

Outcome	Crude OR [95% CI]	*p*-Value	Adjusted OR ‡ [95% CI]	*p*-Value
NICU admission	0.933 [0.494–1.761]	0.830	1.168 (0.557–2.450)	0.681
Prematurity	1.268 [0.734–2.192]	0.395	0.946 (0.505–1.774)	0.864
LBW	1.806 [1.054–3.095]	0.031	1.464 (0.781–2.743)	0.234
APGAR score < 7 at 5 min	2.938 [0.908–9.510]	0.072	1.719 (0.464–6.375)	0.418

‡ OR after adjustment for maternal age.

## Data Availability

The datasets used and analyzed during the current study are available from the corresponding author upon reasonable request.
